# Dairy Consumption and Insulin Resistance: The Role of Body Fat, Physical Activity, and Energy Intake

**DOI:** 10.1155/2015/206959

**Published:** 2015-01-29

**Authors:** Larry A. Tucker, Andrea Erickson, James D. LeCheminant, Bruce W. Bailey

**Affiliations:** College of Life Sciences, Brigham Young University, Provo, UT 84602, USA

## Abstract

The relationship between dairy consumption and insulin resistance was ascertained in 272 middle-aged, nondiabetic women using a cross-sectional design. Participants kept 7-day, weighed food records to report their diets, including dairy intake. Insulin resistance was assessed using the homeostatic model assessment (HOMA). The Bod Pod was used to measure body fat percentage, and accelerometry for 7 days was used to objectively index physical activity. Regression analysis was used to determine the extent to which mean HOMA levels differed across low, moderate, and high dairy intake categories. Results showed that women in the highest quartile of dairy consumption had significantly greater log-transformed HOMA values (0.41 ± 0.53) than those in the middle-two quartiles (0.22 ± 0.55) or the lowest quartile (0.19 ± 0.58) (*F* = 6.90, *P* = 0.0091). The association remained significant after controlling for each potential confounder individually and all covariates simultaneously. Adjusting for differences in energy intake weakened the relationship most, but the association remained significant. Of the 11 potential confounders, only protein intake differed significantly across the dairy categories, with those consuming high dairy also consuming more total protein than their counterparts. Apparently, high dairy intake is a significant predictor of insulin resistance in middle-aged, nondiabetic women.

## 1. Introduction

Increasing rates of overweight and obesity worldwide have generated concern about a diabetes epidemic, with associated negative effects on quality of life, life expectancy, and healthcare costs. Recent data from the United States suggests that about 26 million people (8.3% of the population) are affected by diabetes [1], with more than 90% of these suffering from type 2 diabetes mellitus [2]. Danaei et al. reported that the number of individuals with diabetes worldwide has nearly doubled over the past 30 years [3]. The substantial economic and healthcare burdens placed on society by type 2 diabetes mellitus (T2DM) demonstrate a need for improved prevention efforts, particularly given its largely avertable nature.

To better control T2DM, considerable effort has been devoted to research aimed at isolating the determinants of this widespread disorder. To date, many modifiable risk factors of T2DM have been identified [4–7]. Of the various contributors, diet has become a primary focus [8, 9]. Consumption of a healthy diet, commonly characterized by sensible intakes of unsaturated fats and fiber, as well as low intakes of saturated and trans fats and foods with a high glycemic load, has been associated with a decreased risk of developing T2DM [6, 10, 11].

Several studies have also investigated the impact of milk and dairy products on the development of T2DM. Most epidemiological investigations have identified an inverse relationship between dairy consumption, as part of an overall healthy diet, and T2DM [12–14] and the metabolic syndrome [15–17]. However, conflicting results have surfaced [18–22], leaving the relationship inconclusive.

The natural disease progression of T2DM is characterized by the inability of the body to respond to consumption of a glycemic load with the appropriate amount of insulin to mediate glucose uptake [23, 24]. This is known as insulin resistance. Insulin resistance precedes T2DM and is strongly related to obesity and cardiovascular disease [25, 26].

Milk and dairy products have been identified as potent insulin secretagogues, as their consumption stimulates acute hyperinsulinemia [27–31]. The hyperinsulinemia resulting from milk and dairy consumption may be considered a beneficial and even protective effect for regulating blood glucose levels, particularly in individuals with elevated levels or those with T2DM [32]. However, consumption of milk and dairy products and the resultant hyperinsulinemia may produce less-than-desirable long-term effects in healthy individuals, including insulin resistance. Research in humans [33] and in rats [34] suggests that regular hyperinsulinemia can lead to insulin resistance.

Prevention of T2DM is probably best achieved by avoiding the development of insulin resistance. Several modifiable risk factors of insulin resistance have been identified [35–38], among which diet plays a principal role [37, 38]. Unfortunately, the dairy and insulin resistance relationship has not been extensively investigated [39–42], and results have been contradictory.

Measurement method shortcomings are common in studies that have investigated the role of milk and dairy products on disease outcomes. To date, body weight has largely been self-reported or the body mass index (BMI) has been used to estimate body composition. Both of these strategies result in considerable measurement error and frequent misclassification [43]. In addition, questionnaires have been used almost exclusively to assess physical activity levels. Unfortunately, self-reported physical activity is known to be highly biased and contain significant error [44, 45]. Lastly, the vast majority of investigations designed to study the relationship between diet and insulin resistance or T2DM have measured diet using food frequency questionnaires or the 24-hour recall method. Research shows that subjects struggle to recall precisely what they have consumed in the past, and additional error is introduced when subjects are required to estimate portion sizes [46–48]. Energy intake is commonly underreported using these methods [48].

The present study was designed to overcome these measurement deficiencies. A high quality measurement method, air-displacement plethysmography (Bod Pod), was employed to evaluate body fat, rather than body weight. Moreover, physical activity was measured objectively using accelerometers, rather than relying on self-reported estimates of activity. Further, diet and energy intake were evaluated using 7 days of weighed food records.

In conclusion, studies designed to examine the relationship between dairy intake and insulin resistance are sparse, and few investigations have adjusted adequately for differences in body fat, physical activity, diet, energy intake, and other potential confounding factors measured using high quality methods. Research on the association between dairy intake and insulin resistance, using high quality measurement methods, is clearly warranted.

## 2. Methods

### 2.1. Design

The relationship between dairy intake and insulin resistance in a sample of 272 middle-aged women was studied using a cross-sectional design. In addition, determining the extent to which age, weight, body fat percentage, total energy intake, physical activity level, education, grams consumed of carbohydrate, protein, and fat, and insoluble and soluble fiber intake influenced the relationship between dairy consumption and insulin resistance was an ancillary purpose of the investigation.

### 2.2. Participants

Potential subjects were recruited by word-of-mouth and through newspaper advertisements and e-mails circulated to individuals and companies in approximately 20 cities in the Mountain West, United States. Initial inclusion screening was conducted via telephone and focused on recruiting individuals who were female, premenopausal, not pregnant, nondiabetic, nonsmokers, and apparently healthy. Informed consent was obtained from each subject prior to study commencement and was approved by the University's Institutional Review Board.

### 2.3. Procedures

Subject information and measurements were gathered at the university. Measurements and training instructions lasted 60 to 90 minutes. Height and weight were measured for each participant during the initial appointment while wearing a one-piece, lab-issued swimsuit. While wearing the same swimsuit and a swim cap, a Bod Pod test (Life Measurements Instruments, Concord, CA) was performed on each subject to estimate body fat percentage. Subjects were taught how to accurately measure food intake using a digital food scale (Ohaus 2000, Florham Park, NJ) and were instructed to keep a seven-day weighed food record. A nine-page diet log, including specific directions for recording dietary intake, a sample page, and blank records for each day of the week, was given to each subject. Each subject was asked to read the instructions. Common recording mistakes were explained to subjects to improve detail and compliance. Next, each subject was given written and verbal instructions regarding the proper way to weigh food with the Ohaus 2000 portable electronic scale using plastic food models.

Each subject was issued an Actigraph accelerometer (Health One Technology, formerly CSA, Pensacola, FL), which they were instructed to wear continuously over the left hip for seven consecutive days, with the exception of bathing or water events. Participants were encouraged to maintain their normal lifestyle and to avoid implementing new dietary or exercise practices. Explanations of proper techniques were provided to all participants so that they understood correct procedures.

During the 7-day period, participants were contacted by study personnel by telephone to ensure that they were accurately recording everything consumed and that they were maintaining a typical diet and physical activity levels and to answer questions. Participants were given a blood requisition form, which they took to a local hospital during the seven days, following a 12-hour fast, to have their blood drawn by lab phlebotomists. At the end of the 7 days, subjects returned the food record, food scale, and accelerometer. Subjects were weighed again wearing a one-piece lab-issued swimsuit. An average of the two body weight measures allowed body weight to be indexed based on the average of two measures taken a week apart, rather than one assessment. Once it was determined that the data obtained was accurate and complete, subjects were mailed a thank you letter with a $25 gift certificate.

### 2.4. Instrumentation and Measurements

The criterion variable for this study was insulin resistance, assessed using the homeostatic model assessment (HOMA). The primary predictor variable was servings of dairy foods, which were measured using 7-day weighed food records. Partial correlation was used to determine the extent to which potential confounding variables, namely, age, education, total energy intake, multiple dietary variables, objectively measured physical activity level, and body fat percentage, affected the dairy consumption and insulin resistance relationship.

*Insulin Resistance*. Lab phlebotomists withdrew a blood sample from the antecubital vein after the subjects had fasted for at least 12 hours. Drinking water during the 12 hours was allowed. The samples were stored at about −20°C after being centrifuged for 15 minutes at 2000 g at 4°C. The Access Ultrasensitive Insulin assay (Beckman Coulter, Brea, CA) was used to determine fasting insulin (*μ*U/L). Dimension Vista System and Flex reagent cartridges (Siemens, Deerfield, IL) were used to measure fasting glucose levels (mg/dL). HOMA [49] was used to assess insulin resistance using the following formula: fasting plasma insulin (*μ*U/mL) × fasting plasma glucose (mg/dL)/405. HOMA has been shown to be comparable to the euglycemic clamp as a means of assessing insulin resistance (*r* = 0.82, *P* = 0.0002) and is considered valid and reliable [49, 50]. 

*Dietary Intake*. Seven-day weighed food records were used to measure dairy consumption, total energy intake, carbohydrate, protein, and fat consumption, and insoluble and soluble fiber intake. This method minimizes subject recall bias and effectively represents an individual's normal dietary patterns by covering weekdays and weekends [47, 48]. Weighed food records have frequently been employed as a standard for comparison when assessing the validity of other dietary intake measurements [51, 52], and seven days has been shown to be an appropriate length of time to accurately assess intake [47, 48].

Subjects were issued a digital food scale (Ohaus 2000, Florham Park, NJ) and were instructed how to properly weigh and record foods and beverages using plastic food models and verbal directions. Printed instructions were also given to each participant. Subjects were taught about the importance of maintaining a typical diet over the week of recording. To help combat the tendency to underreport food consumption [48], participants were informed that they would be weighed before and after the week of recording and were asked to not gain or lose weight during the week. If a participant's food record indicated her daily intake was less than 130% of her estimated resting metabolic rate, determined using the Ravussin formula [53], she was required to repeat the weighed food record. Completed food records were returned following the 7-day recording period and were examined for accuracy. A registered dietician input all food records into the ESHA Research software (ESHA Research Inc., Salem, OR, USA) for further analysis.

Dietary analysis categorized dairy intake based on the American Dietetic Association (now Academy of Nutrition and Dietetics) and American Diabetes Association (ADA) Exchange Lists program. In the Exchange Lists program, a fat-free/low-fat serving of dairy is defined as 12 g of carbohydrate, 8 g of protein, and 0–3 g of fat. In the present study, typical fat-free/low-fat dairy foods included fat-free milk, 1/2% milk, 1% milk, low-fat buttermilk, evaporated fat-free milk, low fat soy milk, and fat-free yogurt, including those with artificial sweeteners. A serving of reduced-fat dairy foods included those with 12 g carbohydrate, 8 g of protein, and 5 g of fat. Specific foods included 2% milk, soy milk, sweet acidophilus milk, and plain low-fat yogurt. Similarly, one serving of whole dairy was defined as 12 g of carbohydrate, 8 g protein, and 8 g of fat. In the present study, specific foods included whole milk, evaporated whole milk, goat's milk, kefir, and yogurt made with whole milk. The American Dietetic and American Diabetes Associations (ADA) Exchange Lists do not include cheese in the dairy category because the macronutrient composition of cheese differs significantly from other dairy foods, as defined above. Differences in fat across the dairy categories, fat free/low-fat, reduced fat, and whole dairy, were controlled in the present study, so that each dairy serving had the same energy content, as used in other studies [54, 55]. Partial servings were calculated to within 0.1 servings. 

*Physical Activity*. Physical activity was assessed using Actigraph accelerometers, model 7164 (Health One Technology, Fort Walton Beach, FL, USA). Accelerometers are superior to self-reported physical activity, which is known to be biased and contain significant error [44, 45]. Many investigations have been conducted to validate the Actigraph in adults, showing a close representation between the physical activity levels of free living subjects and doubly labeled water and portable metabolic systems [56–58].

A pilot study testing 15 women from the present investigation evaluated the reliability of the accelerometers as they took part in seventeen different activities, such as walking, jogging, and stair climbing at different speeds and grades. The same assessments were performed one week following the baseline tests. The test-retest intraclass reliability for each activity was greater than 0.90 and was greater than 0.98 for the sum of the seventeen activities.

Physical activity was measured objectively for 7 consecutive days using the accelerometer. Other than during bathing or water activities, the accelerometer was worn constantly throughout the day and night. The accelerometer was worn over the left hip, attached to a nylon belt that was worn around the waist. Following the testing period, participants returned the accelerometers and investigators downloaded their activity data and checked for accuracy. Any participant who failed to wear the accelerometer for at least 12 hours during waking hours was required to re-wear it for the corresponding day(s) of the week as the noncompliant day(s). Final data included 7 days of valid wear time for every subject. Average wear time from 7 A.M. to 10 P.M., a 15-hour period, was 13.9 hours (93% wear-time compliance) across the 7 consecutive days.

In the present study, total physical activity was indexed using the sum of all the activity counts acquired over the seven days of assessment. Concurrent validity for this measure has been shown by several investigations [59–62]. 

*Body Fat Percentage*. Air-displacement plethysmography (Bod Pod) was used to estimate body fat percentage (Life Measurements Instruments, Concord, CA). The Bod Pod was also used to assess thoracic lung volume, which was subtracted from body volume. Subjects were instructed to fast and avoid exercise for at least three hours before their appointment, according to standard protocol. They were given a lab-issued swimsuit and a swim cap to complete the test in and were asked to void, if possible, before the assessment. Body composition was measured in the Bod Pod at least twice. If the body fat percentage results differed by more than one percentage point, then another measurement was taken. This process was repeated until two results were within one percentage point, and then the average of these two outcomes was used to index body fat percentage.

The Bod Pod has been shown to be valid and reliable in estimating body fat percentage. Maddalozzo et al. [63] demonstrated concurrent validity for the Bod Pod compared to dual energy X-ray absorptiometry. Concurrent validity of the body fat percentage measure resulting from the Bod Pod and dual energy X-ray absorptiometry was also established with a sample of 100 women from the current study, with an intraclass correlation of 0.97 (*P* < 0.0001) [64]. In addition, test-retest using the Bod Pod and the same sample of 100 women resulted in an intraclass correlation of 0.99 (*P* < 0.0001) [65]. Estimating body fat percentage with the Bod Pod is a much more valid strategy than using BMI, as BMI often produces misclassification of overweight and obesity [43].

### 2.5. Statistical Analysis

Statistical analysis was conducted using the SAS software program, version 9.3 (Cary, NC). Because HOMA values deviated from a normal distribution, they were log-transformed. To simplify reading and to avoid redundancy, log-transformed HOMA values are referred to as HOMA throughout the paper. Bivariate associations were determined using Pearson correlations. The extent to which mean HOMA levels differed across categories of dairy intake was determined using regression analysis and the General Linear Model (GLM) procedure. For these computations, dairy intake was divided into quartiles and the middle-two quartiles were combined forming three categories: low (0 to 0.5 servings of dairy per day), moderate (0.6 to 1.5 servings of dairy per day), and high (1.6 to 6 servings of dairy per day). Dairy intake was also calculated as servings per 4184 kJ (1000 kcal), forming the following categories: low (0 to 0.23 servings per 4184 kJ (1000 kcal)), moderate (0.24 to 0.79 servings per 4184 kJ (1000 kcal)), and high (0.80 to 3.1 servings per 4184 kJ (1000 kcal)). To examine the influence of specific potential confounders, such as age, education, body weight, energy intake, diet, body fat percentage, and physical activity, considered individually and collectively, on the relationship between dairy consumption and HOMA, partial correlations were computed using the GLM procedure. Adjusted means were calculated using the least-squares means procedure.

A power analysis was conducted using the PASS 6.0 statistical software (NCSS, Kaysville, UT, USA) to determine the number of participants needed to achieve 0.80 power with alpha set at 0.05 when evaluating mean differences across three categories (low, moderate, and high) using ANOVA to detect a small effect size of 0.20. Results showed that 240 subjects would be sufficient. Overall, with more than 270 participants, the study had excellent power.

## 3. Results

A total of 272 women participated in the present investigation. Subjects were primarily Caucasian (~90%), middle-aged (40.1 ± 3.0 years), working either full- or part-time (58%), and married (82%), and about 32% had at least some college education.  Table 1 shows additional descriptive characteristics for the study participants, including total physical activity, body fat percentage, weight, fasting glucose, fasting insulin, total energy intake, percent of energy from carbohydrate, protein, and fat, insoluble and soluble fiber intakes per 4184 kJ (1,000 kilo-calories), average servings of dairy consumed per day, HOMA, and log-transformed HOMA. Means, standard deviations, minimum and maximum values, and quartiles are also displayed in Table 1. Average dairy intake for these women was 1.1 ± 1.0 servings per day. Women with low consumption (bottom quartile) averaged 0.2 ± 0.2 servings of dairy per day, while the moderate category had 1.0 ± 0.4 serving per day, and the high dairy participants (top quartile) had 2.4 ± 0.9 servings per day. Mean servings per 4184 kJ (1000 kcal) was 0.6 ± 0.5. Average HOMA was 1.5 ± 1.0 and mean log-transformed HOMA was 0.3 ± 0.6.

Table 2 shows mean differences in the various potential confounding variables across the three dairy consumption categories, low, moderate, and high, including age, body weight, body fat percentage, energy intake, objectively measured physical activity, carbohydrate and fat intake, and fiber consumption, insoluble and soluble. None of these measures differed across the dairy intake categories. However, grams of protein intake per day differed significantly across the dairy categories. Specifically, women with high dairy intake had higher protein intake than those in the moderate or low dairy consumption categories (*F* = 7.57, *P* = 0.0006).

Table 3 displays mean differences in HOMA across the three dairy consumption categories, without and with adjustment for the potential confounders. As shown, when no variables were controlled, significant differences in mean HOMA were seen across the three dairy consumption categories (*F* = 6.90, *P* = 0.0091). Those in the high dairy consumption category had significantly higher HOMA levels (0.41 ± 0.53) than those in the moderate (0.22 ± 0.55) or low consumption categories (0.19 ± 0.58). Differences in the potential confounding factors, including age, weight, body fat percentage, energy intake, total physical activity, education, carbohydrate, protein, and fat consumption, insoluble fiber intake, and soluble fiber intake, considered individually or collectively, failed to influence appreciably the relationship between dairy consumption and HOMA (Table 3).

Specifically, the relationship was attenuated slightly after controlling for age (*F* = 6.77, *P* = 0.0098), education (*F* = 6.48, *P* = 0.0114), and percent of calories from protein (*F* = 5.87, *P* = 0.0160), yet it remained statistically significant. Adjusting for differences in energy intake weakened the relationship by 32% (*F* = 4.68, *P* = 0.0315). Controlling for several other variables strengthened the relationship, including body weight (*F* = 9.18, *P* = 0.0027), body fat percentage (*F* = 7.67, *P* = 0.0060), total physical activity (*F* = 7.47, *P* = 0.0067), percent of calories from fat (*F* = 8.40, *P* = 0.0041), intake of insoluble fiber (*F* = 7.45, *P* = 0.0068), and intake of soluble fiber (*F* = 7.69, *P* = 0.0059). After controlling for all of the potential confounding factors simultaneously, the dairy and HOMA relationship was weakened, but remained significant (*F* = 4.71, *P* = 0.0309) (Table 2).

With dairy consumption expressed as servings per 4184 kJ (1000 kcal), results were generally weaker, but all models remained statistically significant. With no variables controlled (*F* = 5.30, *P* = 0.0220), women with high dairy intake per 4184 kJ (1000 kcal) had significantly higher HOMA levels (0.39 ± 0.54) than those in the moderate (0.23 ± 0.56) or low consumption categories (0.19 ± 0.55). Likewise, after adjusting statistically for differences in all of the potential confounders (*F* = 5.30, *P* = 0.0223), women with high dairy intake had significantly higher HOMA levels (0.35 ± 0.54) than women in the moderate (0.20 ± 0.56) or low categories (0.16 ± 0.55), calculated as servings per 4184 (1000 kcal).

The Pearson associations between HOMA and the potential confounders including age (*r* = 0.02, *P* = 0.7515), physical activity (*r* = −0.09, *P* = 0.1201), percent of total calories from carbohydrate (*r* = −0.09, *P* = 0.1562), protein (*r* = 0.07, *P* = 0.2367), and fat (*r* = 0.06, *P* = 0.3238), and insoluble fiber intake (*r* = −0.06, *P* = 0.3045) were not statistically significant. However, there were significant bivariate relationships between HOMA and body fat percentage (*r* = 0.47, *P* < 0.0001), body weight (*r* = 0.39, *P* < 0.0001), fasting plasma glucose (*r* = 0.45, *P* < 0.0001), fasting plasma insulin (*r* = 0.91, *P* < 0.0001), total energy intake (*r* = 0.23, *P* < 0.0001), and soluble fiber intake (*r* = −0.17, *P* = 0.0040).

## 4. Discussion

In the present study, there was a significant and meaningful relationship between dairy consumption, assessed using 7-day weighed diet records, and insulin resistance, measured using HOMA. Specifically, women with high dairy intake (top 25%) had significantly greater insulin resistance than women with moderate or low dairy intake (Table 3). The difference between the upper and lower quartiles produced an effect size of 0.40. The association remained significant after controlling statistically for several potential confounding variables, including age, body weight, body fat percentage, energy intake, total physical activity, education, percent of energy from carbohydrate, protein or fat, insoluble fiber intake, and soluble fiber consumption, considered individually or collectively. Adjusting for differences in energy intake had the strongest effect on the association, but it remained significant. The association also remained statistically significant, with and without control of the potential confounding variables, when dairy consumption was expressed as servings per 4184 kJ (1000 kcal).

Although a very different sample, the present findings are in line with an intervention by Hoppe et al. [40] who studied 24 eight-year-old boys in 2005. HOMA increased significantly after one week in those given a dairy supplement, but did not change in those given a meat supplement. The researchers did not state that subjects were randomly assigned to groups. The groups seemed to differ on factors other than the milk and meat intervention. It does not appear that energy intake, body composition, or physical activity levels were controlled.

Also in agreement with the present study were findings from Snijder et al. [66] who found that higher dairy consumption was significantly associated with higher fasting glucose levels in a sample from the Netherlands, where dairy consumption is generally high. In addition, Lawlor et al. [67] examined the relationship between milk consumption and insulin resistance and the metabolic syndrome in 4,024 British women. It was observed that women who never drank milk had lower HOMA levels, were less likely to have T2DM, and were less likely to manifest the metabolic syndrome than women who drank milk regularly. Milk consumption was measured nominally (yes milk intake versus no milk intake), thus preventing the determination of a dose-response relationship.

Contradicting the present results, Rideout et al. [42] observed that HOMA levels improved in overweight or obese subjects with higher dairy consumption (4 servings per day of milk or yogurt) compared to lower dairy intake (fewer than 2 servings per day of milk or yogurt) over the course of 12 months in a small crossover trial using 23 adults. Participants were free-living and without energy restriction. Body fat was assessed using dual energy X-ray absorptiometry.

Akter et al. [41] found results conflicting with the present study in a cross-sectional investigation of 496 Japanese adults, where higher intake of full-fat milk or yogurt was associated with lower HOMA. Body composition was indexed and controlled using BMI, physical activity was assessed using a self-reported questionnaire, and dietary intake was assessed using a food frequency questionnaire. Akter et al. [41] point out that this population, in general, consumes considerably less dairy than Western populations, and that only a small percentage regularly consume low-fat or fat-free dairy.

Each of the potential confounding variables influenced the relationship between dairy intake and insulin resistance, some more than others. Adjusting for differences in total daily energy intake weakened the relationship by 32%, more than any other variable. Post hoc analyses showed that energy intake was significantly and positively related to dairy intake (*r* = 0.21, *P* = 0.0006), physical activity (*r* = 0.16, *P* = 0.0101), body weight (*r* = 0.40, *P* < 0.0001), and HOMA (*r* = 0.23, *P* < 0.0001). Thus, women with higher energy intakes were more likely to have higher consumption of dairy products, participate in greater amounts of physical activity, have higher body weights, and be more insulin resistant than women with lower energy intakes.

Adjusting for differences in body fat also weakened the dairy—insulin resistance association. Specifically, if all the women of the present study would have had the same level of body fat, the relationship between dairy and insulin resistance would have been 13% weaker (Table 2). In general, investigations of the relationship between dairy consumption and insulin resistance have largely relied on BMI to index body composition and obesity. Unfortunately, this method tends to produce significant error [43]. Obesity is a strong contributor to insulin resistance [68], even in nondiabetic populations [69]. Consequently, the present study assessed body composition to estimate and control for differences in body fat, rather than using BMI.

Physical activity is another important variable that strongly influences insulin sensitivity and therefore was controlled in the present investigation. It is widely accepted that participation in physical activity reduces risk of insulin resistance and T2DM [70, 71]. Both chronic physical activity and single bouts of exercise have been shown to improve insulin sensitivity [72]. To date, no investigation of the relationship between dairy intake and insulin resistance has measured physical activity objectively and controlled for differences among participants. Questionnaires are typically administered to gather physical activity information. However, subject responses to activity questionnaires are often highly skewed [44, 45]. Consequently, the present study employed accelerometry over a period of seven days to objectively assess each subject's engagement in physical activity. Adjusting for differences in physical activity had little influence on the dairy—insulin resistance relationship of the present study, strengthening it by only 7%, however.

Other variables that strengthened the relationship between dairy consumption and HOMA were body weight (strengthened by 12%), percent of energy from carbohydrate (strengthened by 13%), percent of energy derived from dietary fat (strengthened by 21%), insoluble fiber intake (strengthened by 8%), and soluble fiber intake (strengthened by 8%).

Consistent with the present findings, it has been shown in the literature that dietary fiber intake is associated with improved insulin sensitivity [73–75], particularly higher intake of soluble fiber [74]. There was a significant positive relationship between soluble fiber intake and physical activity (*r* = 0.15, *P* = 0.0114), but soluble fiber was negatively associated with HOMA (*r* = −0.17, *P* = 0.0040). These relationships may partly explain why controlling for soluble fiber intake strengthened the association between dairy consumption and insulin resistance. Namely, women who ate more soluble fiber also participated in greater amounts of physical activity and had lower HOMA levels.

Diets categorized by consistently high glycemic loads tend to predict insulin resistance and subsequent T2DM in both men [76] and women [10], since chronically high insulin requirements to mediate glucose uptake can lead to reduced insulin sensitivity over time. Therefore, consumption of diets with a low glycemic index is recommended to prevent T2DM. Dairy is considered to have a relatively low glycemic index [77], inferring that it may not adversely affect insulin requirements. However, the insulinemic index has been shown to be three to six times higher than expected based on the glycemic index of dairy foods [28, 30], suggesting that there is an insulinotropic component in milk products [28–31]. Thus, while it has been established that chronic hyperglycemia can lead to insulin resistance [78], research indicates that chronic hyperinsulinemia may also lead to reduced insulin sensitivity [33, 34].

High intake of animal protein has been linked to increased risk of T2DM [79]. Elevated levels of amino acids interfere with normal glucose metabolism, particularly in individuals with reduced insulin sensitivity, leading to insulin resistance [79–81]. High consumption of dairy protein could exacerbate insulin resistance. As shown in Table 2, women with high dairy consumption had significantly higher total intakes of protein, which could help explain why those with high dairy intake had the highest level of insulin resistance in the present study.

Beta cell function should be taken into account when discussing insulin secretion. Persistent consumption of foods categorized by either a high glycemic index or a high insulinemic index causes beta cells to release more insulin to initiate glucose uptake into body cells, leading to insulin insensitivity [10, 76]. According to Leahy et al. [78] and Polonsky et al. [82], this could lead to reduced insulin sensitivity and eventual T2DM, as pancreatic beta cells hypersecrete insulin to maintain normal blood glucose levels, leading to beta cell failure, a key feature of T2DM [78, 82].

The hyperinsulinemic response associated with dairy consumption [27–31] may be considered a beneficial and even protective effect for regulating blood glucose levels, particularly in individuals with T2DM [32]. However, this does not mean that the effects of chronic milk and dairy intake on insulin levels in healthy individuals necessarily follow a similar pattern. Similarly, perhaps the short-term benefits of milk and dairy consumption for blood glucose regulation produce adverse long-term effects, including reduced insulin sensitivity.

As is the case with all cross-sectional research, reverse causality is a potential threat. Although the strong relationship between dairy intake and insulin resistance may be a result of dairy consumption causing hyperinsulinemia, leading to insulin resistance over time, it is also possible that women with elevated blood glucose levels chose to consume more dairy, possibly to help control their unhealthy blood glucose levels. Moreover, other factors, “third variables,” could be responsible for the relationship between dairy and insulin resistance. However, because about a dozen possible confounding variables were controlled in the present study, the link between dairy and insulin resistance is not likely a function of one of these factors, but other variables not controlled in the present study could account for the results.

An important strength of the present study was its use of high quality, objective measurement methods to control for several potential confounding variables. The Bod Pod was used to estimate body composition rather than BMI. Accelerometry was used to assess physical activity instead of a questionnaire, and 7-day weighed food records were used to quantify dietary intake instead of a questionnaire, thereby reducing the problems associated with diet recall and estimation of serving sizes.

The present investigation was not without weaknesses, however. It was limited by its cross-sectional design, thus preventing the establishment of a cause-and-effect relationship. Also, the focus of the study was on nondiabetic, middle-aged, nonsmoking women, and the sample was largely homogeneous, predominately Caucasian women. Overweight and obesity were not common in the present sample. Hence, generalization of the findings may be limited to populations with similar characteristics.

## 5. Conclusion

The present study uncovered a significant relationship between dairy consumption and reduced insulin sensitivity in middle-aged, nondiabetic women, suggesting that higher intakes of dairy products may be associated with greater insulin resistance. This relationship was partly explained by differences in body composition, body weight, physical activity, dietary fiber intake, and energy consumption, particularly the latter. However, high dairy consumption remained a significant predictor of insulin resistance after adjusting for all covariates. If a causal relationship was assumed, then high dairy intake may lead to reduced insulin sensitivity over time. Clearly, more research focusing on the relationship between dairy intake and insulin resistance is needed before changes in dairy consumption can be recommended for the prevention of insulin resistance in nondiabetic women.

## Figures and Tables

**Table 1:** Descriptive statistics (*n* = 272).

Variables	Mean	SD	Min	Percentile			Max
				25th	50th	75th	
Weight (kg)	66.1	10.0	42.1	58.9	65.2	72.0	95.5
Age (years)	40.1	3.0	34.0	38.0	40.0	43.0	46.0
Activity (counts/week)^*^	2700.1	781.9	827.8	2103.9	2669.6	3166.6	4945.9
Body fat (%)	31.7	6.9	14.6	27.2	32.2	36.8	44.8
Fasting glucose (mg/dL)	86.7	5.9	73.0	82.0	87.0	90.0	111.0
Fasting insulin (*μ*U/mL)	7.0	4.2	1.2	4.3	6.1	8.3	34.8
Energy intake (kJ/day)	8585.1	1335.0	6293.7	7624.0	8386.4	9332.0	14623.0
Energy intake (kcal/day)	2051.9	319.1	1504.0	1822.1	2004.4	2230.4	3495.1
Carbohydrate (% of total kJ)	55.7	6.2	25.4	51.7	56.0	59.4	73.3
Protein (% of total kJ)	13.8	2.5	8.5	12.3	13.5	15.1	25.5
Fat (% of total kJ)	30.5	5.8	11.6	27.1	30.3	34.5	51.6
Insoluble fiber (g/4184 kJ)^†^	3.8	1.9	0.5	2.5	3.4	4.7	12.6
Soluble fiber (g/4184 kJ)^†^	1.7	0.9	0.2	1.1	1.6	2.0	6.3
Dairy intake (serv./day)	1.1	1.0	0.0	0.5	1.0	1.6	6.0
Dairy intake (serv./4184 kJ)	0.6	0.5	0.0	0.2	0.5	0.8	3.1
HOMA^‡^	1.5	1.0	0.2	0.9	1.3	1.8	8.3
HOMA (log-transformed)	0.3	0.6	−1.5	−0.1	0.3	0.6	2.1

^*^Average activity counts for 1 week objectively measured using accelerometers, divided by 1000.

^†^Fiber intake is expressed as g per 4184 kJ (1000 kcal).

^‡^HOMA, homeostasis model assessment of insulin resistance.

**Table 2:** Mean differences in the potential confounders across the dairy intake categories.

HOMA	Dairy consumption categories						*F*	*P*
	Low consumption		Moderate consumption		High consumption			
	*n* = 68		*n* = 136		*n* = 68			
	Mean	SD	Mean	SD	Mean	SD		
Age (years)	39.8	3.2	40.0	2.9	40.7	3.0	1.94	0.1455
Weight (kg)	65.8	11.1	66.8	9.3	64.9	10.2	0.83	0.4352
Body fat (%)	31.8	7.0	31.6	7.0	31.8	6.8	0.04	0.9568
Energy intake (kJ/day)	482.4	85.0	493.7	69.0	491.9	80.2	0.52	0.5958
Physical activity (counts)^*^	275.2	79.2	266.8	80.8	271.3	72.4	0.28	0.7590
Carbohydrate intake (g)	278.2	65.5	293.6	48.1	295.7	51.4	2.32	0.1000
Protein intake (g)	68.0^a^	16.9	70.8^a^	13.9	77.5^b^	14.4	7.57	0.0006
Fat intake (g)	73.7	17.8	71.5	17.3	66.9	19.9	2.53	0.0814
Insoluble fiber (g/4184 kJ)^‡^	3.8	2.3	3.6	1.6	4.1	2.0	1.90	0.1512
Soluble fiber (g/4184 kJ)^‡^	1.7	0.9	1.6	0.9	1.7	0.8	0.22	0.8040

^*^Activity counts were divided by 10,000 to make the values more manageable. An activity count of 275.2 means that the group had 2.752 million activity counts for the week.

^‡^Fiber intake is expressed as grams per 4184 kJ (g per 1000 kcal), as is dairy consumption.

None of the results were statistically significant except protein intake. Means on the same row with different superscript letters were significantly different (*P* < 0.05).

Low consumption included women with dairy intake at or below the 25th percentile. Moderate consumption included those whose dairy intake was between the 25th and 75th percentiles. High consumption included those with dairy intake at or above the 75th percentile. Mean dairy consumption for the low, moderate, and high consumption categories were 0.2 ± 0.2, 1.0 ± 0.4, and 2.4 ± 0.9 servings per day, respectively.

Because “education” was a categorical variable, the relationship between dairy intake and education was analyzed using Chi-square. The results showed no association between the two variables (*P* = 0.4524).

**Table 3:** Mean differences in HOMA by the dairy intake categories, without and with adjustment for the potential confounders.

HOMA^*^	Dairy consumption categories						*F*	*P*
	Low consumption		Moderate consumption		High consumption			
	*n* = 68		*n* = 136		*n* = 68			
	Mean	SD	Mean	SD	Mean	SD		
Variable controlled								
None	0.19^a^	0.58	0.22^a^	0.55	0.41^b^	0.53	6.90	0.0091
Age (years)	0.19^a^		0.22^a^		0.41^b^		6.77	0.0098
Weight (kg)	0.21^a^		0.21^a^		0.43^b^		9.18	0.0027
Body fat (%)	0.18^a^		0.23^a^		0.40^b^		7.67	0.0060
Energy intake (kJ/day)	0.22^a†^		0.22^a^		0.39^b^		4.68	0.0315
Total activity (counts/week)	0.19^a^		0.22^a^		0.42^b^		7.47	0.0067
Education	0.18^a^		0.21^a^		0.40^b^		6.48	0.0114
Carbohydrate (% of kJ)	0.17^a^		0.23^a^		0.42^b^		7.84	0.0055
Protein intake (% of kJ)	0.19^a^		0.23^a^		0.41^b^		5.87	0.0160
Fat intake (% of kJ)	0.17^a^		0.23^a^		0.43^b^		8.40	0.0041
Insoluble fiber (g/4184 kJ)	0.19^a^		0.22^a^		0.42^b^		7.45	0.0068
Soluble fiber (g/4184 kJ)	0.19^a^		0.22^a^		0.42^b^		7.69	0.0059
All covariates	0.17^a†^		0.19^a^		0.34^b^		4.71	0.0309

^*^HOMA values were log-transformed.

^†^Statistically significant at the trend level (0.05 < *P* < 0.10).

Means on the same row with the same superscript letter are not significantly different (*P* > 0.05).

Low consumption included women with dairy intake at or below the 25th percentile. Moderate consumption included dairy intake between the 25th and 75th percentiles. High consumption included dairy intake at or above the 75th percentile. Mean dairy consumption (servings per 4184 kJ) for the low, moderate, and high consumption categories were 0.1 ± 0.1, 0.5 ± 0.2, and 1.2 ± 0.4 servings per day, respectively.

## References

[1] CDC (2012). *Crude and Age-Adjusted Rate Percentage of Civilian, Noninstitutionalized Adults with Diagnosed Diabetes, United States, 1980–2011*.

[2] Zimmet P., Alberti K. G. M. M., Shaw J. (2001). Global and societal implications of the diabetes epidemic. *Nature*.

[3] Danaei G., Finucane M. M., Lu Y. (2011). National, regional, and global trends in fasting plasma glucose and diabetes prevalence since 1980: systematic analysis of health examination surveys and epidemiological studies with 370 country-years and 2.7 million participants. *The Lancet*.

[4] Eriksson K.-F., Lindgarde F. (1991). Prevention of type 2 (non-insulin-dependent) diabetes mellitus by diet and physical exercise. The 6-year Malmö feasibility study. *Diabetologia*.

[5] Gillies C. L., Abrams K. R., Lambert P. C. (2007). Pharmacological and lifestyle interventions to prevent or delay type 2 diabetes in people with impaired glucose tolerance: systematic review and meta-analysis. *British Medical Journal*.

[6] Hu F. B., Manson J. E., Stampfer M. J. (2001). Diet, lifestyle, and the risk of type 2 diabetes mellitus in women. *The New England Journal of Medicine*.

[7] Knowler W. C., Barrett-Connor E., Fowler S. E. (2002). Reduction in the incidence of type 2 diabetes with lifestyle intervention or metformin. *The New England Journal of Medicine*.

[8] West K. M., Kalbfleisch J. M. (1971). Influence of nutritional factors on prevalence of diabetes. *Diabetes*.

[9] van Dam R. M., Rimm E. B., Willett W. C., Stampfer M. J., Hu F. B. (2002). Dietary patterns and risk for type 2 diabetes mellitus in U.S. men. *Annals of Internal Medicine*.

[10] Salmerón J., Manson J. E., Stampfer M. J., Colditz G. A., Wing A. L., Willett W. C. (1997). Dietary fiber, glycemic load, and risk of non-insulin-dependent diabetes mellitus in women. *Journal of the American Medical Association*.

[11] Schulze M. B., Liu S. M., Rimm E. B., Manson J. E., Willett W. C., Hu F. B. (2004). Glycemic index, glycemic load, and dietary fiber intake and incidence of type 2 diabetes in younger and middle-aged women. *The American Journal of Clinical Nutrition*.

[12] Choi H. K., Willett W. C., Stampfer M. J., Rimm E., Hu F. B. (2005). Dairy consumption and risk of type 2 diabetes mellitus in men—a prospective study. *Archives of Internal Medicine*.

[13] Liu S., Choi H. K., Ford E. (2006). A prospective study of dairy intake and the risk of type 2 diabetes in women. *Diabetes Care*.

[14] Malik V. S., Sun Q., van Dam R. M. (2011). Adolescent dairy product consumption and risk of type 2 diabetes in middle-aged women. *The American Journal of Clinical Nutrition*.

[15] Elwood P. C., Pickering J. E., Fehily A. M. (2007). Milk and dairy consumption, diabetes and the metabolic syndrome: the Caerphilly prospective study. *Journal of Epidemiology and Community Health*.

[16] Fumeron F., Lamri A., Abi Khalil C. (2011). Dairy consumption and the incidence of hyperglycemia and the metabolic syndrome. Results from a French prospective study, data from the epidemiological study on the insulin resistance syndrome (DESIR). *Diabetes Care*.

[17] Pereira M. A., Jacobs D. R., van Horn L., Slattery M. L., Kartashov A. I., Ludwig D. S. (2002). Dairy consumption, obesity, and the insulin resistance syndrome in young adults: the CARDIA study. *The Journal of the American Medical Association*.

[18] Kirii K., Mizoue T., Iso H. (2009). Calcium, vitamin D and dairy intake in relation to type 2 diabetes risk in a Japanese cohort. *Diabetologia*.

[19] Pittas A. G., Dawson-Hughes B., Li T. (2006). Vitamin D and calcium intake in relation to type 2 diabetes in women. *Diabetes Care*.

[20] Sluijs I., Forouhi N. G., Beulens J. W. J. (2012). The amount and type of dairy product intake and incident type 2 diabetes: results from the EPIC-InterAct study. *The American Journal of Clinical Nutrition*.

[21] Snijder M. B., van Dam R. M., Stehouwer C. D. A., Hiddink G. J., Heine R. J., Dekker J. M. (2008). A prospective study of dairy consumption in relation to changes in metabolic risk factors: the Hoorn study. *Obesity*.

[22] van Dam R. M., Hu F. B., Rosenberg L., Krishnan S., Palmer J. R. (2006). Dietary calcium and magnesium, major food sources, and risk of type 2 diabetes in U.S. black women. *Diabetes Care*.

[23] Defronzo R. A., Bonadonna R. C., Ferrannini E. (1992). Pathogenesis of NIDDM—a balanced overview. *Diabetes Care*.

[24] Alberti K. G., Zimmet P. Z. (1998). Definition, diagnosis and classification of diabetes mellitus and its complications. Part 1: diagnosis and classification of diabetes mellitus provisional report of a WHO consultation. *Diabetic Medicine*.

[25] DeFronzo R. A., Ferrannini E. (1991). Insulin resistance: a multifaceted syndrome responsible for NIDDM, obesity, hypertension, dyslipidemia, and atherosclerotic cardiovascular disease. *Diabetes Care*.

[26] Reaven G. M. (1988). Role of insulin resistance in human disease. *Diabetes*.

[27] Hoppe C., Mølgaard C., Dalum C., Vaag A., Michaelsen K. F. (2009). Differential effects of casein versus whey on fasting plasma levels of insulin, IGF-1 and IGF-1/IGFBP-3: results from a randomized 7-day supplementation study in prepubertal boys. *European Journal of Clinical Nutrition*.

[28] Gannon M. C., Nuttall F. Q., Krezowski P. A., Billington C. J., Parker S. (1986). The serum insulin and plasma glucose responses to milk and fruit products in type 2 (non-insulin-dependent) diabetic patients. *Diabetologia*.

[29] Hoyt G., Hickey M. S., Cordain L. (2005). Dissociation of the glycaemic and insulinaemic responses to whole and skimmed milk. *British Journal of Nutrition*.

[30] Östman E. M., Elmståhl H. G. M. L., Björck I. M. E. (2001). Inconsistency between glycemic and insulinemic responses to regular and fermented milk products. *The American Journal of Clinical Nutrition*.

[31] Liljeberg Elmståhl H., Björck I. (2001). Milk as a supplement to mixed meals may elevate postprandial insulinaemia. *European Journal of Clinical Nutrition*.

[32] Nuttall F. Q., Gannon M. C. (1988). Quantiative importance of dietary constituents other than glucose as insulin secretagogues in type II diabetes. *Diabetes Care*.

[33] del Prato S., Leonetti F., Simonson D. C., Sheehan P., Matsuda M., DeFronzo R. A. (1994). Effect of sustained physiologic hyperinsulinaemia and hyperglycaemia on insulin secretion and insulin sensitivity in man. *Diabetologia*.

[34] Juan C.-C., Fang V. S., Kwok C.-F., Perng J.-C., Chou Y.-C., Ho L.-T. (1999). Exogenous hyperinsulinemia causes insulin resistance, hyperendothelinemia, and subsequent hypertension in rats. *Metabolism: Clinical and Experimental*.

[35] Yokoyama H., Hirose H., Ohgo H., Saito I. (2004). Associations among lifestyle status, serum adiponectin level and insulin resistance. *Internal Medicine*.

[36] Kahn B. B., Flier J. S. (2000). Obesity and insulin resistance. *The Journal of Clinical Investigation*.

[37] Ryan A. S. (2000). Insulin resistance with aging—effects of diet and exercise. *Sports Medicine*.

[38] Barnard R. J., Youngren J. F., Martin D. A. (1995). Diet, not aging, causes skeletal muscle insulin resistance. *Gerontology*.

[39] Hirschler V., Oestreicher K., Beccaria M., Hidalgo M., Maccallini G. (2009). Inverse association between insulin resistance and frequency of milk consumption in low-income Argentinean school children. *The Journal of Pediatrics*.

[40] Hoppe C., Mølgaard C., Vaag A., Barkholt V., Michaelsen K. F. (2005). High intakes of milk, but not meat, increase s-insulin and insulin resistance in 8-year-old boys. *European Journal of Clinical Nutrition*.

[41] Akter S., Kurotani K., Nanri A. (2013). Dairy consumption is associated with decreased insulin resistance among the Japanese. *Nutrition Research*.

[42] Rideout T. C., Marinangeli C. P. F., Martin H., Browne R. W., Rempel C. B. (2013). Consumption of low-fat dairy foods for 6 months improves insulin resistance without adversely affecting lipids or bodyweight in healthy adults: a randomized free-living cross-over study. *Nutrition Journal*.

[43] Rothman K. J. (2008). BMI-related errors in the measurement of obesity. *International Journal of Obesity*.

[44] Laporte R. E., Montoye H. J., Caspersen C. J. (1985). Assessment of physical activity in epidemiologic research—problems and prospects. *Public Health Reports*.

[45] Tucker J. M., Welk G. J., Beyler N. K. (2011). Physical activity in U.S.: adults compliance with the physical activity guidelines for Americans. *American Journal of Preventive Medicine*.

[46] Guthrie H. A. (1984). Selection and quantification of typical food portions by young adults. *Journal of the American Dietetic Association*.

[47] Hartman A. M., Brown C. C., Palmgren J. (1990). Variability in nutrient and food intakes among older middle-aged men. Implications for design of epidemiologic and validation studies using food recording. *The American Journal of Epidemiology*.

[48] Baranowski T., Willett W. C. (2013). 24-Hour recall and diet record methods. *Nutritional Epidemiology*.

[49] Matthews D. R., Hosker J. P., Rudenski A. S., Naylor B. A., Treacher D. F., Turner R. C. (1985). Homeostasis model assessment: insulin resistance and *β*-cell function from fasting plasma glucose and insulin concentrations in man. *Diabetologia*.

[50] Bonora E., Saggiani F., Targher G. (2000). Homeostasis model assessment closely mirrors the glucose clamp technique in the assessment of insulin sensitivity: studies in subjects with various degrees of glucose tolerance and insulin sensitivity. *Diabetes Care*.

[51] Bingham S. A., Cassidy A., Cole T. J. (1995). Validation of weighed records and other methods of dietary assessment using the 24-h urine nitrogen technique and other biological markers. *British Journal of Nutrition*.

[52] Jain M., Howe G. R., Rohan T. (1996). Dietary assessment in epidemiology: comparison of a food frequency and a diet history questionnaire with a 7-day food record. *American Journal of Epidemiology*.

[53] Ravussin E., Lillioja S., Anderson T. E., Christin L., Bogardus C. (1986). Determinants of 24-hour energy expenditure in man—methods and results using a respiratory chamber. *The Journal of Clinical Investigation*.

[54] Tucker L. A., Tucker J. M., Bailey B., LeCheminant J. D. (2014). Meat intake increases risk of weight gain in women: a prospective cohort investigation. *American Journal of Health Promotion*.

[55] Tucker L. A., Tucker J. M., Bailey B., LeCheminant J. D. (2014). Dietary patterns as predictors of body fat and BMI in women: a factor analytic study. *The American Journal of Health Promotion*.

[56] Bassett D. R., Ainsworth B. E., Swartz A. M., Strath S. J., O'Brien W. L., King G. A. (2000). Validity of four motion sensors in measuring moderate intensity physical activity. *Medicine and Science in Sports and Exercise*.

[57] Brage S., Wedderkopp N., Franks P. W., Bo Andersen L., Froberg K. (2003). Reexamination of validity and reliability of the CSA monitor in walking and running. *Medicine and Science in Sports and Exercise*.

[58] Liu A.-L., Li Y.-P., Song J., Pan H., Han X.-M., Ma G.-S. (2005). Study on the validation of the computer science application's activity monitor in assessing the physical activity among adults using doubly labeled water method. *Zhonghua liu xing bing xue za zhi*.

[59] Tucker L. A., Tucker J. M. (2011). Television viewing and obesity in 300 women: evaluation of the pathways of energy intake and physical activity. *Obesity*.

[60] Tucker L. A., Peterson T. R. (2003). Objectively measured intensity of physical activity and adiposity in middle-aged women. *Obesity Research*.

[61] Nokes N. R., Tucker L. A. (2012). Changes in hip bone mineral density and objectively measured physical activity in middle-aged women: a 6-year prospective study. *American Journal of Health Promotion*.

[62] LeCheminant J., Tucker L., Russell K. (2011). Physical activity and C-reactive protein levels: the confounding role of body fat. *Journal of Physical Activity and Health*.

[63] Maddalozzo G. F., Cardinal B. J., Snow C. M. (2002). Concurrent validity of the BOD POD and dual energy X-ray absorptiometry techniques for assessing body composition in young women. *Journal of the American Dietetic Association*.

[64] LeCheminant J. D., Tucker L. A., Peterson T. R., Bailey B. W. (2001). Differences in body fat percentage measured using dual energy X-Ray absorptiometry and the Bod Pod in 100 women. *Medicine & Science in Sports & Exercise*.

[65] Tucker L. A., Lecheminant J. D., Bailey B. W. (2014). Test-retest reliability of the BOD POD: the effect of multiple assessments. *Perceptual and Motor Skills*.

[66] Snijder M. B., van der Heijden A. A. W. A., van Dam R. M. (2007). Is higher dairy consumption associated with lower body weight and fewer metabolic disturbances? The Hoorn study. *The American Journal of Clinical Nutrition*.

[67] Lawlor D. A., Ebrahim S., Timpson N., Smith G. D. (2005). Avoiding milk is associated with a reduced risk of insulin resistance and the metabolic syndrome: findings from the British Women's Heart and Health Study. *Diabetic Medicine*.

[68] Ludvik B., Nolan J. J., Baloga J., Sacks D., Olefsky J. (1995). Effect of obesity on insulin resistance in normal subjects and patients with NIDDM. *Diabetes*.

[69] Abbasi F., Brown B. W., Lamendola C., McLaughlin T., Reaven G. M. (2002). Relationship between obesity, insulin resistance, and coronary heart disease risk. *Journal of the American College of Cardiology*.

[70] Helmrich S. P., Ragland D. R., Leung R. W., Paffenbarger R. S. (1991). Physical activity and reduced occurrence of non-insulin-dependent diabetes mellitus. *The New England Journal of Medicine*.

[71] Ivy J. L. (1997). Role of exercise training in the prevention and treatment of insulin resistance and non-insulin-dependent diabetes mellitus. *Sports Medicine*.

[72] Borghouts L. B., Keizer H. A. (2000). Exercise and insulin sensitivity: a review. *International Journal of Sports Medicine*.

[73] Rave K., Roggen K., Dellweg S., Heise T., Dieck H. T. (2007). Improvement of insulin resistance after diet with a whole-grain based dietary product: results of a randomized, controlled cross-over study in obese subjects with elevated fasting blood glucose. *British Journal of Nutrition*.

[74] Breneman C. B., Tucker L. A. (2013). Dietary fibre consumption and insulin resistance—the role of body fat and physical activity. *British Journal of Nutrition*.

[75] Ludwig D. S., Pereira M. A., Kroenke C. H. (1999). Dietary fiber, weight gain, and cardiovascular disease risk factors in young adults. *Journal of the American Medical Association*.

[76] Salmerón J., Ascherio A., Rimm E. B. (1997). Dietary fiber, glycemic load, and risk of NIDDM in men. *Diabetes Care*.

[77] Björck I., Liljeberg H., Östman E. (2000). Low glycaemic-index foods. *British Journal of Nutrition*.

[78] Leahy J. L., Bonner-Weir S., Weir G. C. (1992). *β*-cell dysfunction induced by chronic hyperglycemia: current ideas on mechanism of impaired glucose-induced insulin secretion. *Diabetes Care*.

[79] Sluijs I., Beulens J. W. J., van der A D. L., Spijkerman A. M. W., Grobbee D. E., van der Schouw Y. T. (2010). Dietary intake of total, animal, and vegetable protein and risk of type 2 diabetes in the European prospective investigation into cancer and nutrition (EPIC)-NL study. *Diabetes Care*.

[80] Promintzer M., Krebs M. (2006). Effects of dietary protein on glucose homeostasis. *Current Opinion in Clinical Nutrition and Metabolic Care*.

[81] Tremblay F., Lavigne C., Jacques H., Marette A. (2007). Role of dietary proteins and amino acids in the pathogenesis of insulin resistance. *Annual Review of Nutrition*.

[82] Polonsky K. S., Sturis J., Bell G. I. (1996). Non-insulin-dependent diabetes mellitus—a genetically programmed failure of the beta cell to compensate for insulin resistance. *The New England Journal of Medicine*.

